# Association between Life’s Crucial 9 and psoriatic arthritis in U.S. adults: a cross-sectional study

**DOI:** 10.3389/fmed.2025.1574896

**Published:** 2025-08-07

**Authors:** Jiefeng Jiang, Lyuxin Guan, Ziqin Gan, Zhili Wu, Xiaofeng Liang, Zhiwen Zhang, Yansi Lyu, Yunsheng Liang

**Affiliations:** ^1^The First School of Clinical Medicine, Southern Medical University, Guangzhou, China; ^2^Dermatology Hospital, Southern Medical University, Guangzhou, China; ^3^Department of Burn and Plastic Surgery, The First Affiliated Hospital of Shenzhen University, Shenzhen, China; ^4^Department of Dermatology, The Fifth Affiliated Hospital of Sun Yat-sen University, Zhuhai, China; ^5^Department of Dermatology, Shenzhen University General Hospital, Shenzhen, China

**Keywords:** cardiovascular health, Life’s Crucial 9, NHANES, psoriasis, psoriatic arthritis

## Abstract

**Background:**

Psoriatic arthritis (PsA) has been closely associated with an elevated risk of cardiovascular disease (CVD). Nevertheless, the connection between Life’s Crucial 9 (LC9), which serves as a holistic measure of cardiovascular health (CVH), and PsA remains insufficiently studied. This research aims to explore the potential relationship between LC9 and the prevalence of PsA.

**Objective:**

To investigate the role of LC9 in PsA and explore its implications.

**Methods:**

This study utilized a cross-sectional, population-based design, analyzing data from 7,531 U.S. participants aged 20 years or older, drawn from the 2005–2006 and 2009–2014 cycles of the National Health and Nutrition Examination Survey (NHANES). The LC9 score, which encompasses nine distinct components, was classified into three CVH categories: low, moderate, and high. To explore the association between LC9 scores and the prevalence of PsA, logistic regression models and restricted cubic splines were applied.

**Results:**

In the analyzed cohort of 7,531 individuals, participants with moderate or high CVH levels demonstrated a markedly reduced likelihood of developing PsA relative to those with low CVH. Additionally, a 10-point elevation in the LC9 score was associated with a 32% lower odds of having PsA. A notable interaction between LC9 and age was detected. Conversely, no meaningful link was found between LC9 and psoriasis without arthritis (PsC).

**Conclusion:**

The LC9 score and its subcomponents are significantly negatively correlated with the risk of PsA. This suggests that adherence to the lifestyle defined by LC9 is associated with a lower prevalence of PsA.

## Introduction

Psoriasis, an immune-related chronic condition, presents with erythematous plaques usually covered with adherent, silvery-white scales that may be associated with pruritus. In more advanced stages, it may involve the joints. This condition has been associated with an increased risk of heart diseases, diabetes, and metabolic issues, which can greatly diminish a person’s overall well-being (1). PsA, a chronic inflammatory musculoskeletal disorder associated with psoriasis, is a condition that can severely affect a person’s daily life and contributes to long-term morbidity. It occurs in approximately 17.58% of psoriasis patients (2). In addition to physical comorbidities, PsA patients also experience a high psychological burden, including depression and anxiety, which may interact with cardiovascular health (3).

PsA is closely associated with a heightened risk of cardiovascular disease, driven by systemic inflammation, metabolic comorbidities, and shared risk factors such as dyslipidemia, hypertension, and obesity. The Life’s Essential 8 (LE8), crafted by the American Heart Association, serves as a metric for assessing CVH. This tool gauges CVH by considering factors including diet, nicotine exposure, physical activity, sleep, body mass index, blood lipids, blood pressure, and blood glucose. Higher LE8 scores represent better CVH. Obesity has been shown to be an independent risk factor for psoriasis according to Takeshita et al. (4), and factors such as poor dietary habits, smoking, less physical activity, and higher BMI have been associated with the onset of psoriasis according to Shen et al. (5). Zhang et al. (6) in a study investigating LE8 scores and the prevalence of psoriasis in 9,876 American adults aged 20–59 years showed a significant negative correlation between LE8 scores and the risk of developing psoriasis. In addition, a prospective study of Ouyang et al. (7) followed 261,642 participants who did not have psoriasis at baseline for an average of up to 12 years to assess the relationship between their LE8 score and the prevalence of psoriasis. At the end of follow-up, 1,501 participants had psoriasis, with a lower prevalence in those with moderate and high LE8 scores. In conclusion, a current research of Zhang et al. (6) indicated an inverse relationship between the LE8 score and the likelihood of developing psoriasis. Given the substantial cardiovascular burden in PsA patients, identifying and addressing modifiable lifestyle factors becomes a critical aspect of disease prevention and management (8–10).

While the LE8 is widely recognized as a comprehensive tool for assessing CVH, it does not include mental health, despite increasing acknowledgment of its critical role in cardiovascular outcomes. To bridge this gap, the LC9 was introduced as an extension of LE8, incorporating a ninth component—depression—to better capture the influence of psychological well-being on CVH (11). Although some studies have suggested that LC9 may not markedly improve CVH risk prediction over LE8 (12), the inclusion of mental health is particularly relevant in the context of PsA, a condition often accompanied by psychological distress, including depression and anxiety (3). Indeed, individuals with PsA exhibit a significantly higher prevalence of depressive symptoms compared to those with PsC or the general population (13). Notably, the American Heart Association has formally recognized psychological health as a fundamental pillar of CVH (11), further supporting the epidemiological and clinical rationale for incorporating depression into CVH metrics. Therefore, LC9 may offer a more holistic and sensitive measure of cardiovascular health in populations with a substantial mental health burden, such as individuals with PsA. In the present study, we employed LC9 to examine its association with PsA using data from a nationally representative sample of U.S. Adults.

## Method

For this research, we examined data collected during the National Health and Nutrition Examination Survey (NHANES) spanning the periods of 2005–2006 and 2009–2014. To assess the correlations, we employed a multivariable logistic regression model. We conducted subgroup analyses focusing on variables such as gender, age, ethnicity, poverty-to-income ratio (PIR), educational attainment, and relationship status to gain a deeper understanding of the link between LC9 and PsA. The dataset for our study is accessible to the public via the NHANES online portal.

### Study population

The National Health and Nutrition Examination Survey (NHANES) aims to assess the incidence of significant health conditions and their related risk factors among Americans. For a comprehensive overview of the survey’s methodologies and objectives, refer to https://www.cdc.gov/nchs/nhanes/index.html. NHANES utilizes a sophisticated, multiphase, probability-based approach for sampling to guarantee a sample that mirrors the national demographic. The research protocol of NHANES received clearance from the Institutional Review Board at the National Center for Health Statistics (NCHS), with all subjects offering their informed written consent prior to participation. As our study is based on data that is both accessible to the public and stripped of personal identifiers, there was no necessity for further ethical clearance or participant consent (14).

**Figure 1:**
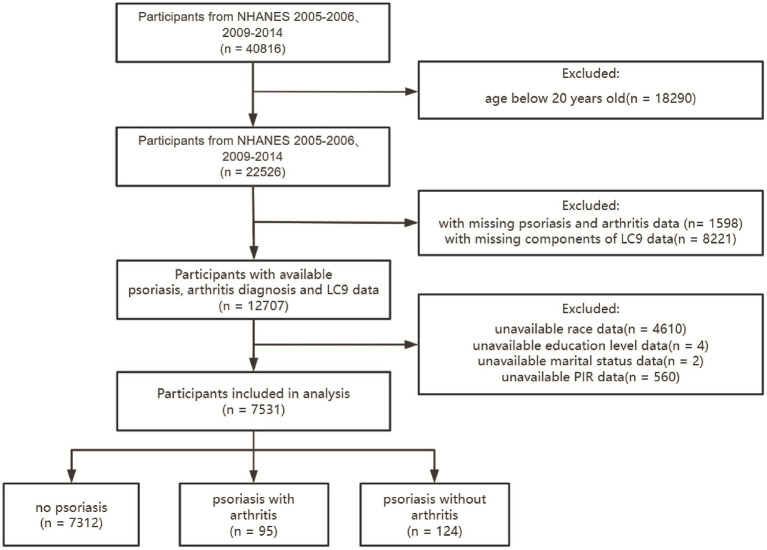
Flowchart.

In this cross-sectional research, we analyzed data gathered during the 2005–2006 and 2009–2014 periods of the NHANES, adhering to the guidelines set by STROBE (Strengthening the Reporting of Observational Studies in Epidemiology). Our study focused on individuals who were 20 years of age or older and had comprehensive information regarding their LC9 scores, psoriasis, and arthritis. We excluded those who lacked essential demographic details, including race, marital status, education level or poverty-to-income ratio. Out of the 40,816 participants from the specified NHANES cycles, 7,531 were eligible based on our criteria. A flowchart detailing participant selection and inclusion is provided in Figure 1.

### Measurements of LC9

The LC9 score is determined as the mean value of nine components, comprising four health behaviors (nicotine exposure, diet, sleep duration, and physical activity) and four health factors (blood glucose, body mass index, blood pressure, and non-high-density lipoprotein cholesterol), alongside a depression score.

The algorithm for calculating the LC9 score has been previously detailed in the literature. Briefly, each CVH indicator is quantified on a 0 to 100 measurement scale, with scores assigned by an expert panel using a modified Delphi method based on their association with health outcomes and risks. The composite LC9 score is thus the mean of these individual assessments (11). Participants were categorized into three CVH levels: high CVH (LC9 scores of 80 or higher), moderate CVH (scores ranging from 50 to 79), and low CVH (scores below 50), consistent with the classification method for LE8 (15).

Assessment of the nutritional aspect of LC9 is conducted with the aid of the Healthy Eating Index from 2015 (HEI-2015) (16). Information on food consumption is gathered via two 24-h dietary recall interviews and is then integrated with food pattern data sourced from the USDA to determine the HEI-2015 score.

Sleep duration, nicotine exposure, diabetes history, physical activity, and medication history were gathered via standardized self-reported questionnaires. During the physical examination, blood pressure, weight, and height were measured. The Body Mass Index (BMI) was derived by computing the individual’s weight in kilograms divided by the height in meters, squared. Blood samples were extracted for subsequent analysis at a central laboratory, where hemoglobin A1c levels, fasting blood glucose, and lipid profiles were assessed.

The depression score is based on the Patient Health Questionnaire-9 (PHQ-9), a validated tool for screening depressive symptoms. Higher PHQ-9 scores reflect greater severity of current depressive symptoms. Depression scores are categorized as 100, 75, 50, 25, and 0, corresponding to PHQ-9 ranges of 0–4, 5–9, 10–14, 15–19, and 20–27, respectively (17).

### Diagnosis of psoriasis and psoriatic arthritis

Identification of individuals with psoriasis and arthritis was based on their answers to the medical history questionnaire, using the questions: “(Have you/ Has SP) ever been told by a health care provider that (you/he/she) had psoriasis?” and “Has a doctor or other healthcare professional ever told you/has [he/she] ever been told that you have arthritis?” Due to survey limitations, formal diagnostic criteria for PsA could not be applied. Instead, a concurrent diagnosis of both psoriasis and arthritis was used as a proxy for PsA. The study population was compared with two control groups: an “intermediate control group” comprising individuals with PsC and a “normal control group” consisting of participants without psoriasis (18).

### Covariates

In alignment with prior research, this study took into account several variables: sex, age, race, education level, marital status, and household income. In NHANES, race and Hispanic origin were self-reported through questionnaire responses. Participants were sorted into one of four ethnic categories as NHANES classification: non-Hispanic White, non-Hispanic Black, Mexican American, and Other (which includes multiracial individuals and non-Hispanic Asians).

The educational background was broken down into three levels: college graduate or higher, some college, and high school or less. Marital status was simplified into two distinct categories: living alone and married or living with a partner. Economic status was assessed by ranking household income against the poverty threshold, resulting in three brackets: low income (at or below 1.3), middle income (between 1.3 and 3.5), and high income (above 3.5).

### Statistical analysis

An overview of the study’s participants was provided through a descriptive evaluation. The baseline characteristics are expressed as means with standard deviations (SD) for continuous variables, and as frequencies with percentages for categorical variables. To compare baseline characteristics, participants were divided into three groups: PsA, PsC, and individuals without psoriasis. Continuous variables were analyzed using either analysis of variance (ANOVA) or the Kruskal-Wallis test, while categorical variables were evaluated with the chi-square test.

We utilized a multivariable multinomial logistic regression analysis to investigate the relationship between LC9 scores and the conditions of PsA and PsC. The analysis yielded odds ratios and 95% confidence intervals. We developed three distinct models: the initial Model 1 without any adjustments for potential influencing factors; Model 2, which incorporated adjustments for sociodemographic including sex, age (treated as a continuous measure), and race; and the comprehensive Model 3, which built upon Model 2 by further accounting for marital status, education level, and PIR. To validate the continuous variable of the LC9 score, we categorized it similarly to LE8 and calculated the trend *p*-value (19). Moreover, we conducted analyses using multivariable logistic regression to examine the links between specific elements of the LC9 score and the presence of PsA, with considerations for possible confounding elements.

To further investigate the nonlinear relationship, restricted cubic splines (RCS) regression was applied to the LC9 scores to evaluate the dose–response association with PsA and PsC (20).

Subgroup analyses were conducted to examine the relationship between LC9 and PsA in different populations, stratified by sex, age (as a categorical variable), race, education level, marital status, and PIR. Interaction significance was assessed by evaluating the *p*-values for the interaction terms between LC9 and the subgroup categories.

The statistical analysis was performed with the R 4.3.2 programming language (as referenced at http://www.R-project.org, R Foundation). We utilized dual-tailed tests for our assessments, establishing a threshold of *p* < 0.05 to define statistical significance.

## Results

### Population characteristics

This study encompassed 7,531 individuals, with an average age of 48.0 years, featuring a standard deviation of 17.4. The gender distribution revealed that 52.1% of the participants identified as female. Race composition within the sample included 43.5% non-Hispanic White, 22.2% non-Hispanic Black, and 11.1% Mexican American. A notable 59.2% of the participants had attained a college degree or an advanced level of education. The percentage of participants who were married or living with a partner was 59.0%, which was higher than that of those living alone.

Demographic details for the study’s participants are outlined in Table 1. The prevalence of PsA in this study was 1.26%; 1.65% of participants had PsC; and 97.1% had no psoriasis. Among the three study groups, significant baseline differences were observed across all variables, except for sex, PIR, and marital status. Compared to those without psoriasis or with PsC, individuals with PsA tended to be older, more likely to be female, of lower income, with a college education or less, and had a higher tendency to be married or living with partners.

**Table 1:** Baseline characteristics.

Variables	Overall, *N* = 7,531^1^	No psoriasis, *N* = 7,312^1^	With arthritis, *N* = 95^1^	Without arthritis, *N* = 124^1^	*P^2^*
Sex					0.12
Male	3,605 (47.9%)	3,509 (48.0%)	34 (35.8%)	62 (50.0%)	
Female	3,926 (52.1%)	3,803 (52.0%)	61 (64.2%)	62 (50.0%)	
Age (years)	48.0 (17.4)	48.0 (17.4)	60.0 (13.5)	46.0 (16.8)	<0.001
Age group					<0.001
20–39 years	2,477 (32.9%)	2,427 (33.2%)	7 (7.4%)	43 (34.7%)	
40–59 years	2,457 (32.6%)	2,377 (32.5%)	33 (34.7%)	47 (37.9%)	
60+ years	2,597 (34.5%)	2,508 (34.3%)	55 (57.9%)	34 (27.4%)	
Race					0.004
Mexican American	836 (11.1%)	823 (11.3%)	6 (6.3%)	7 (5.6%)	
Non-Hispanic White	3,279 (43.5%)	3,155 (43.1%)	55 (57.9%)	69 (55.6%)	
Non-Hispanic Black	1,672 (22.2%)	1,641 (22.4%)	16 (16.8%)	15 (12.1%)	
Other	1,744 (23.2%)	1,693 (23.2%)	18 (18.9%)	33 (26.6%)	
PIR					0.2
<1.3	2,476 (32.9%)	2,401 (32.8%)	38 (40.0%)	37 (29.8%)	
1.3–3.5	2,628 (34.9%)	2,556 (35.0%)	32 (33.7%)	40 (32.3%)	
>3.5	2,427 (32.2%)	2,355 (32.2%)	25 (26.3%)	47 (37.9%)	
Education level					0.028
High school or less	3,076 (40.8%)	2,995 (41.0%)	40 (42.1%)	41 (33.1%)	
College	2,380 (31.6%)	2,305 (31.5%)	39 (41.1%)	36 (29.0%)	
College graduate or higher	2,075 (27.6%)	2,012 (27.5%)	16 (16.8%)	47 (37.9%)	
Marital status					0.5
Married or living with partners	4,441 (59.0%)	4,319 (59.1%)	50 (52.6%)	72 (58.1%)	
Living alone	3,090 (41.0%)	2,993 (40.9%)	45 (47.4%)	52 (41.9%)	
Health factors score	68.75 (19.96)	68.75 (19.95)	55.00 (17.91)	72.50 (19.19)	<0.001
Health behaviors score	56.25 (18.76)	56.25 (18.82)	56.25 (15.91)	61.25 (17.26)	0.070
Patient Health Questionnaire-9 score	1.00 (4.43)	1.00 (4.39)	3.00 (6.19)	2.00 (4.70)	0.001
Depression score	100 (20)	100 (19)	100 (29)	100 (22)	0.006
LC9	66.67 (13.24)	66.67 (13.22)	57.78 (12.68)	66.67 (12.57)	<0.001
LC9 group					0.001
Low CVH	877 (11.6%)	846 (11.6%)	22 (23.2%)	9 (7.3%)	
Moderate CVH	5,475 (72.7%)	5,311 (72.6%)	69 (72.6%)	95 (76.6%)	
High CVH	1,179 (15.7%)	1,155 (15.8%)	4 (4.2%)	20 (16.1%)	

^1^Mean (SD) for continuous; *n* (%) for categorical. ^2^Pearson’s X^2^: Rao and Scott adjustment; Design-based Kruskal Wallis test. LC9, Life’s Crucial 9; PIR, the ratio of income to poverty.

### Association between LC9 and PsA

Our research investigated the correlation between LC9 scores and the presence of PsA using three models (as detailed in Table 2). The initial Model 1 did not account for any external factors, whereas Model 2 incorporated adjustments for sex, age, and race. Model 3 expanded these adjustments to include education level, household income, and marital status. In the unadjusted Model 1, for every 10-point rise in LC9 score, the probability of PsA occurrence was reduced by 36% (OR = 0.64, 95% CI 0.55–0.75). Incorporating additional factors in Model 2 (OR = 0.97, 95% CI 0.95–0.99) and Model 3 (OR = 0.97, 95% CI 0.95–0.99) led to a modest reduction in the strength of the correlation, yet it was still significant. Furthermore, the categorical LC9 variable analysis revealed that the association persisted across all models. When analyzing Model 3, it was evident that those in the moderate and high CVH categories experienced a diminished risk of PsA onset by 42% (OR = 0.58, 95% CI 0.34–0.99) and 78% (OR = 0.22, 95% CI 0.06–0.83), in contrast to the low CVH group.

**Table 2:** Associations between LC9 and psoriatic arthritis based on multivariable logistic regression models.

LC9	Model 1 OR (95% CI)	Model 2 OR (95% CI)	Model 3 OR (95% CI)
Low CVH	1 (ref)	1 (ref)	1 (ref)
Moderate CVH	0.50 (0.29, 1.85)	0.53 (0.31, 0.91)	0.58 (0.34, 0.99)
High CVH	0.13 (0.04, 0.44)	0.18 (0.05, 0.65)	0.22 (0.06, 0.83)
P for trend	<0.001	0.004	0.012
Per 10-point increase	0.64 (0.55, 0.75)	0.67 (0.56, 0.80)	0.68 (0.57, 0.82)

Model 1, no covariates were adjusted. Model 2, sex, age, and race were adjusted. Model 3, sex, age, race, education level, PIR, marital status were adjusted. LC9, Life’s Crucial 9, PIR, the ratio of income to poverty; OR, Odds Ratio, CI, Confidence Interval.

**Figure 2:**
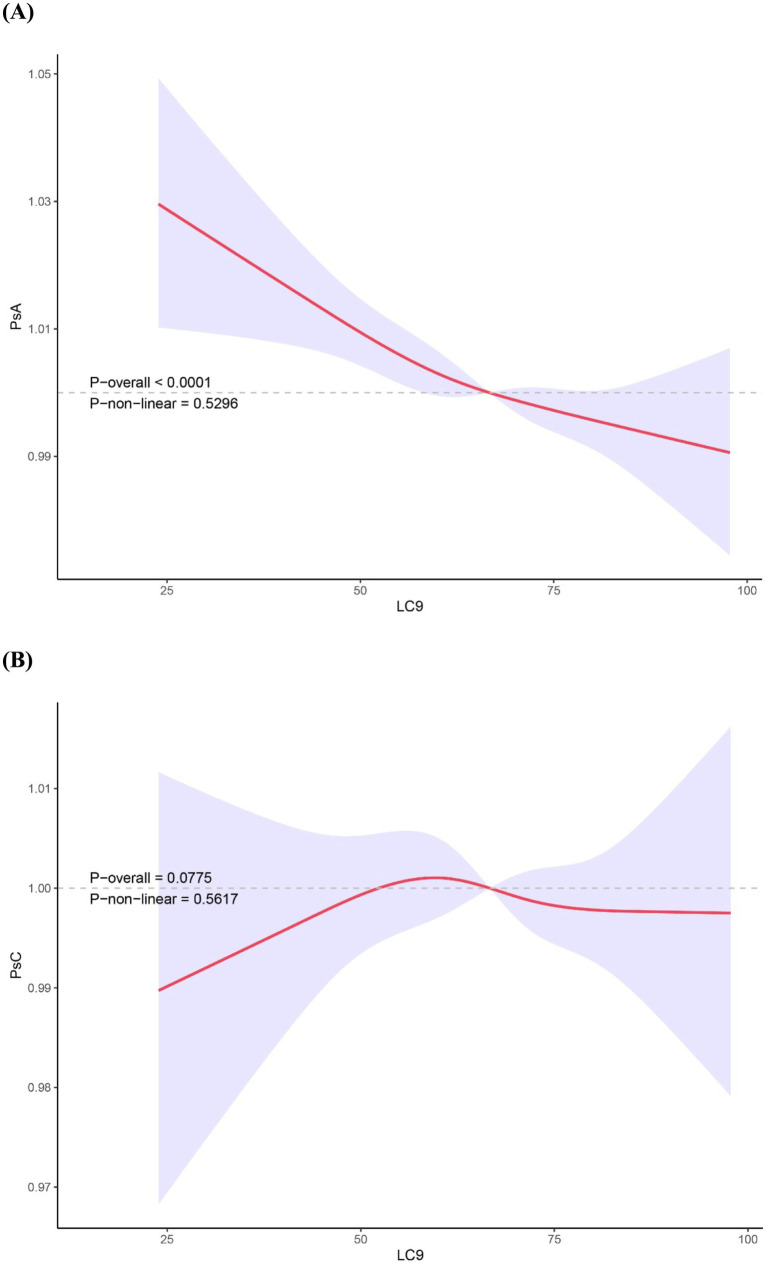
Restricted cubic spline analyses showing the association between LC9 score and the prevalence of PsA **(A)** and PsC **(B)**. ORs (solid lines) and 95% CIs (shaded areas) were adjusted for sex, age (as a continuous variable), race, PIR, education level and marital status. Abbreviation: PsA, Psoriatic arthritis; PsC, psoriasis without arthritis; LC9, Life’s Crucial 9; PIR, the ratio of income to poverty; OR, odds ratio; CI, confidence interval.

A significant dose–response trend was also observed across the three LC9 categories (P for trend = 0.012), indicating a graded inverse relationship between cardiovascular health level and PsA risk. Furthermore, results from multivariate-adjusted restricted cubic spline analysis demonstrated a linear inverse association between the LC9 and PsA (P -overall <0.001, P-non-linear = 0.5296, Figure 2A).

### Association between LC9 and PsC

Our analysis revealed no significant association between LC9 score, when treated as either a continuous or categorical variable, and PsC in any of the three models (Supplementary Table 1). Furthermore, the RCS analysis results confirmed the absence of a non-linear relationship between LC9 and PsC (Figure 2B).

### Association between components of LC9, health behaviors, health factors, depression and PsA

As shown in Table 3, multivariable logistic regression analysis revealed statistically significant inverse associations between PsA and several components of the LC9 score, particularly BMI, blood glucose, health factors score, and the depression score. Furthermore, significant dose–response trends were observed for these components. Higher levels of BMI score (P for trend = 0.016), blood glucose score (P for trend < 0.001), health factors score (P for trend < 0.001) and depression score (P for trend < 0.001) were each associated with lower odds of PsA, indicating graded inverse relationships. These results suggest that, in this cross-sectional analysis, more favorable profiles in specific cardiovascular health domains are correlated with a lower prevalence of PsA.

**Table 3:** Associations between components of LC9, health behaviors, health factors and psoriatic arthritis.

Characteristic	OR^1^	95% CI^1^	*p*-value
^1^HEI-2015 diet score			
Low (0–49)	1 (Reference)		
Moderate (50–79)	0.72	0.42, 1.24	0.223
High (80–100)	0.74	0.39, 1.42	0.353
P for trend			0.309
Per 10-point increase	0.96	0.89, 1.04	0.328
Physical activity score			
Low (0–49)	1 (Reference)		
Moderate (50–79)	1.39	0.57, 3.40	0.447
High (80–100)	0.84	0.56, 1.27	0.393
P for trend			0.427
Per 10-point increase	0.99	0.95, 1.03	0.541
Nicotine exposure score			
Low (0–49)	1 (Reference)		
Moderate (50–79)	1.51	0.80, 2.88	0.193
High (80–100)	0.94	0.56, 1.57	0.811
P for trend			0.292
Per 10-point increase	0.99	0.94, 1.04	0.550
Sleep health score			
Low (0–49)	1 (Reference)		
Moderate (50–79)	0.91	0.56, 1.49	0.694
High (80–100)	0.77	0.45, 1.30	0.305
P for trend			0.283
Per 10-point increase	0.96	0.89, 1.03	0.234
Health behaviors score			
Low (0–49)	1 (Reference)		
Moderate (50–79)	1.05	0.59, 1.85	0.868
High (80–100)	0.45	0.19, 1.04	0.061
P for trend			0.153
Per 10-point increase	0.93	0.83, 1.03	0.133
Body mass index score			
Low (0–49)	1 (Reference)		
Moderate (50–79)	0.81	0.49, 1.34	0.393
High (80–100)	0.36	0.16, 0.82	0.018
P for trend			0.016
Per 10-point increase	0.90	0.83, 0.97	0.004
Blood lipids score			
Low (0–49)	1 (Reference)		
Moderate (50–79)	0.83	0.46, 1.52	0.538
High (80–100)	0.75	0.43, 1.31	0.293
P for trend			0.293
Per 10-point increase	0.94	0.87, 1.02	0.119
Blood glucose score			
Low (0–49)	1 (Reference)		
Moderate (50–79)	0.67	0.39, 1.16	0.143
High (80–100)	0.35	0.21, 0.58	<0.001
P for trend			<0.001
Per 10-point increase	0.88	0.83, 0.93	<0.001
Blood pressure score			
Low (0–49)	1 (Reference)		
Moderate (50–79)	0.73	0.43, 1.26	0.244
High (80–100)	0.58	0.29, 1.14	0.107
P for trend			0.106
Per 10-point increase	0.96	0.89, 1.03	0.187
Health factors score			
Low (0–49)	1 (Reference)		
Moderate (50–79)	0.55	0.34, 0.90	0.019
High (80–100)	0.28	0.12, 0.63	0.004
P for trend			<0.001
Per 10-point increase	0.78	0.69, 0.88	<0.001
Depression score			
Low (0–49)	1 (Reference)		
Moderate (50–79)	0.43	0.21, 0.85	0.018
High (80–100)	0.23	0.10, 0.49	<0.001
P for trend			<0.001
Per 10-point increase	0.84	0.77, 0.92	<0.001

^1^OR, Odds Ratio; CI, Confidence Interval; HEI-2015, Healthy Eating Index from 2015. Adjusted for sex, age, race, education level, and PIR and marital status.

### Subgroup analysis between LC9 and PsA

**Figure 3:**
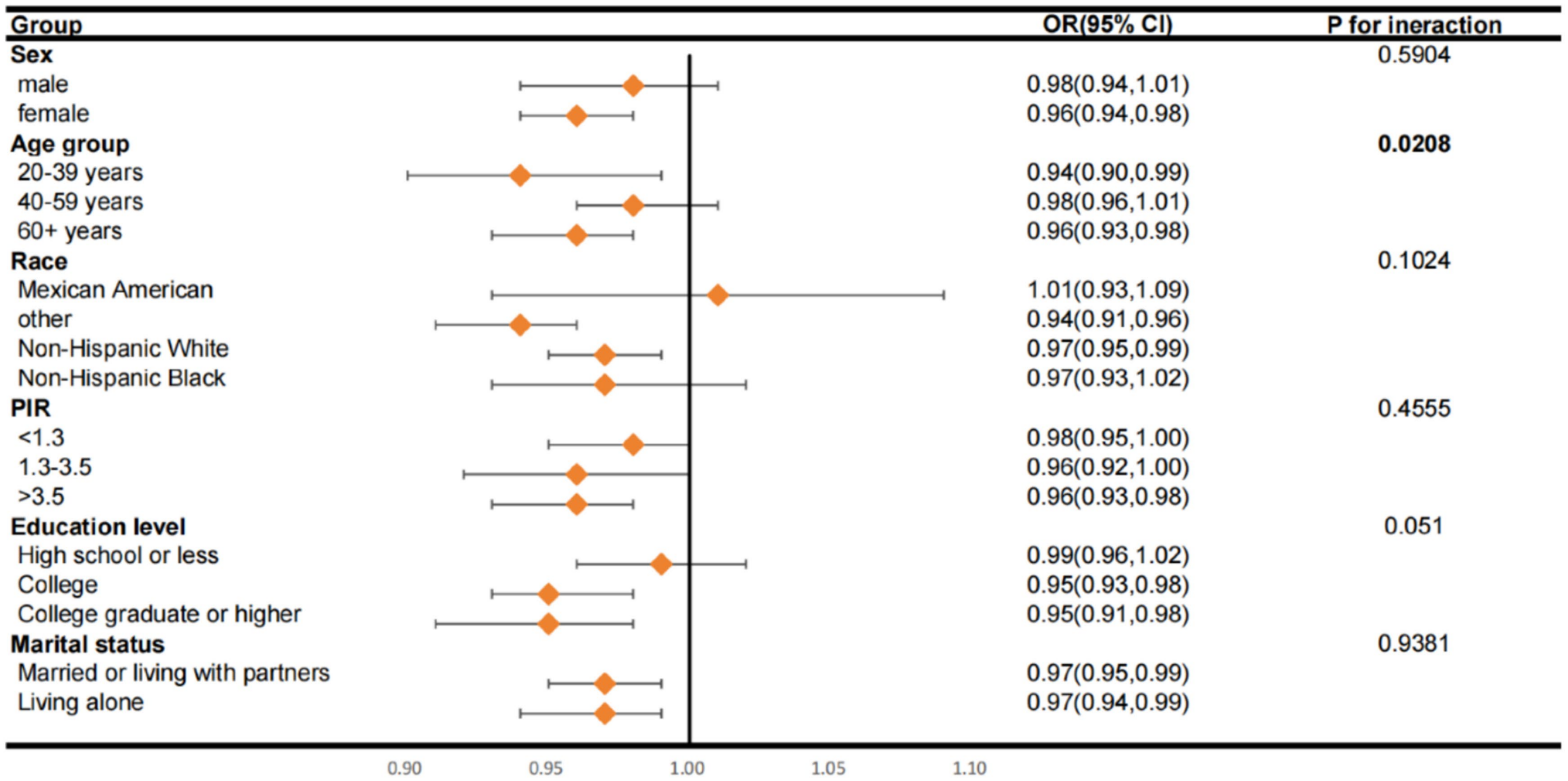
Subgroup analysis of the relationship of Life’s Crucial 9 scores and psoriatic arthritis. ORs were calculated as per score increase in Life’s Crucial 9 total score. Each stratification was adjusted for gender, age (as a categorical variable), race, marital status, education level and family poverty income ratio (as a categorical variable). OR, odds ratio; CI, confidence interval; PIR, family poverty income ratio.

The subgroup analysis results presented in Figure 3 show that, with the exception of Mexican Americans, all other subgroups demonstrated a negative association between LC9 components and PsA. This is in line with our preliminary data, which supports the validity of these observations. Additionally, it was noted that there is a substantial correlation between LC9, age, and the development of PsA, with the interaction being statistically significant (*p* < 0.05).

## Discussion

Within the scope of this cross-sectional study, we analyzed information gathered during the National Health and Nutrition Examination Survey (NHANES), spanning the years 2005–2006 and 2009–2014, to investigate the correlation between PsA and LC9. The results of our study demonstrate that elevated LC9 scores correlate with a lower likelihood of developing PsA. Furthermore, our analysis revealed a pronounced inverse relationship between the composite health factor scores and the risk of PsA. Moreover, higher scores in BMI, blood glucose, and depression were also associated with a lower risk of PsA. This observed inverse association between depression scores and PsA should be interpreted with caution. It is possible that reverse causation is at play—PsA, as a chronic and disabling inflammatory condition, may itself contribute to increased risk of depression. As our study is cross-sectional in nature, it cannot determine the temporal direction of this relationship. Longitudinal studies are needed to clarify whether depressive symptoms contribute to PsA pathogenesis, or if PsA predisposes individuals to poorer mental health. Subgroup analysis showed that, except for the Mexican American subgroup, the negative association between LC9 and PsA was consistent with the overall results.

Psoriasis is a chronic, immune-mediated inflammatory skin disease characterized by scaly erythematous plaques or plaques. As the disease progresses, approximately 17.58% of patients with psoriasis experience PsA. PsA is a persistent inflammatory disease of the joints, whose primary manifestations include joint inflammation, edema, pain, and stiffness, often accompanied by nail damage (2). Psoriasis and PsA, with its chronic nature, not only affects the patient’s physical health, but also extends to the psychological and social aspects of life, posing multifaceted challenges to the patient (21). In the pathogenesis of psoriasis, Th17 and Th23 T-cell subsets are pivotal, which dysregulate pro-inflammatory cytokine synthesis, contributing to overproliferation of epidermal keratinocytes. In the past, psoriasis treatments have focused on Th1 cells, but contemporary research underscores the significance of Th17 cells, which are abundant in the dermis of psoriatic plaques and can trigger dermal inflammation. The IL-23/Th17 signaling pathway is now thought to play an important role in the progression and management of psoriasis (22, 23).

Similarly, PsA was initially considered a Th1-mediated disease; however, current insights reveal that Th17 cells, known for their production of IL-17 and IL-23, play a key role. Among individuals with PsA, there is an elevated count of Th17 cells observed in peripheral blood and within the synovial cavity, with particularly high levels noted in the latter. And these cells exhibit a highly differentiated and multifunctional phenotype. IL-17 induces inflammation and angiogenesis in the synovial tissue, upregulating matrix metalloproteinases in collaboration with other cytokines, and effectively enhancing osteoclast activity. The clinical efficacy of IL-17A antibodies further confirms the critical role of Th17 cells in PsA (24, 25). It is noteworthy that Th17 cells are also pivotal in the pathogenesis of thrombosis and atherosclerosis in cardiovascular diseases (22, 26).

Based on our comprehensive review, this appears to be the inaugural research endeavor to explore the association between PsA and CVH as assessed by the LC9. Our findings show a marked inverse relationship between CVH represented by LC9 scores and the propensity for developing PsA, which is consistent with previous studies (27). Importantly, this inverse relationship is especially evident in the components of BMI, blood glucose, and depression.

Previous research has shown that individuals with PsA exhibit an elevated incidence of risk elements for cardiovascular diseases, encompassing hypertension, dyslipidemia, obesity, diabetes and metabolic syndrome (3, 28). Research has determined that a substantial proportion of PsA cases, specifically 55% of a cohort of 158, presented with arterial hypertension, a condition intricately associated with cardiovascular pathologies, characterized by incidents like coronary artery disease or cerebrovascular ischemia, bearing an odds ratio of 21.0 (29). The role of dyslipidemia in the pathogenesis of PsA remains a subject of ongoing discourse. A contemporary study posits that elevated cholesterol levels could potentially increase the susceptibility to PsA. It has been observed that the likelihood of PsA onset is heightened in those with obesity at the age of 18, irrespective of other contributing factors, with a 5.3% increment in risk for each additional unit of BMI acquired at this age (30, 31). Concurrently, extensive epidemiological studies, one with a sample size of 89,049 American subjects over a 14-year span and another comprising 75,395 individuals with psoriasis in the UK, have identified a correlation between obesity and PsA diagnosis that is contingent on the magnitude of obesity (32, 33). The association between PsA and diabetes mellitus is firmly established. A comprehensive meta-analysis of 557,697 individuals with psoriasis and 5,186,485 non-affected controls has unveiled a correlation between psoriasis and diabetes risk that is contingent on the severity of psoriasis. It was also noted that individuals with PsA exhibited the most significant risk of diabetes (34). Metabolic syndrome (MetS) consists of five major cardiovascular risk factors: including hypertension, hypertriglyceridemia, low HDL-C, abdominal obesity and hyperglycemia. Haroon et al. identified a significant correlation between the severity of PsA and the presence of MetS (35). Notably, studies have revealed that the incidence of MetS is notably greater among individuals diagnosed with PsA compared to those with PsC (36). The U.S. Preventive Services Task Force found that these psychological factors, particularly anxiety and depression, are associated with severe negative health outcomes, including CVD and early CVD mortality (37). Studies have indicated that individuals with PsA are at an increased risk for developing psychiatric comorbidities. Those living with PsA frequently experience disturbances in sleep disorders, fatigue, and low-level stress. It has been observed that the predisposition to depressive disorders is more pronounced in individuals with PsA as compared to those with PsC (13, 38, 39). It is important to highlight that growing evidence indicates a potential link between depression and systemic inflammation, particularly the presence of IL-6, which may contribute to the exacerbation of depressive symptoms. Furthermore, individuals with depression often exhibit elevated levels of CRP and TNF in their blood (39). Studies have also shown that physical activity has a significant positive impact on disease activity, overall well-being, and comorbidities in PsA patients, particularly on metabolic syndrome and obesity. These benefits appear to outweigh the risk of enthesitis caused by mechanical stress (40, 41).

Extensive research has established a robust link among psoriasis, PsA, and CVH. A study has found that higher LC9 scores are associated with a lower risk of psoriasis, and this relationship is partially mediated by systemic inflammation response index (SIRI) (42). While our study did not establish a substantial association between psoriasis and CVH, a pronounced association was observed between PsA and CVH. This could be due to a frequently major severity of the inflammation in PsA compared to psoriasis, which is more likely to be associated with CVH (8). It could also be due to a small and non-representative sample size.

While the exact mechanisms linking LC9 and PsA are not fully understood, a wealth of studies has highlighted the significant roles of lifestyle factors, metabolic syndrome, and mental health in the onset and progression of PsA.

To summarize, our findings suggest that a better cardiovascular health status, as measured by LC9, is associated with a lower prevalence of PsA. Consequently, these results offer valuable insights.

However, this study also has some limitations that need to be considered. First, although adjustments were made for a range of possible confounding factors, the study’s cross-sectional design does not permit the determination of a causal relationship between LC9 scores and the incidence of PsA. Further research is needed to explore the causal relationship over time between LC9 and PsA prevalence. Second, PsA was identified based on self-reported co-occurrence of psoriasis and arthritis, rather than clinical confirmation using validated criteria such as the CASPAR (ClASsification criteria for Psoriatic Arthritis). While this approach allows for analysis within the constraints of the NHANES database, it may introduce misclassification bias. Arthritis of other etiologies could be mistakenly classified as PsA, and recall bias may also contribute to diagnostic inaccuracies. This limitation could reduce the specificity of the outcome definition and should be taken into account when interpreting the associations observed. Third, reliance on self-reported data for certain LC9 elements could introduce biases in the estimates. Fourth, the number of PsA cases in our dataset was relatively small (n = 95), which may have limited the statistical power, particularly in stratified or subgroup analyses. As a consequence, some associations that truly exist may not have reached statistical significance, reflecting the possibility of type II errors. The wide confidence intervals observed in some estimates further underscore the need for cautious interpretation. Fifth, discrepancies in baseline characteristics between included and excluded individuals may confound the interpretation of findings due to missing data at random. Finally, it is also essential to note that the applicability of these findings to a demographically distinct cohort, such as younger adults or those residing in different geographical regions, is yet to be ascertained and warrants additional study.

## Conclusion

There is a potential inverse correlation between the LC9 score and the incidence of PsA among adults in the United States. Among the LC9 components, BMI, blood glucose, and depression scores appear to be particularly important. Collectively, the data suggest that habits and conditions reflected in LC9 are associated with the prevalence of PsA and may be relevant to clinical management. Nonetheless, it is imperative to further investigate the nature of the association between heart health and PsA, including possible underlying mechanisms.

## Data Availability

The original contributions presented in the study are included in the article/Supplementary material, further inquiries can be directed to the corresponding authors.
