# Supplementary material for: Association between Life’s Crucial 9 and psoriatic arthritis in U.S. adults: a cross-sectional study
Source: Front Med (Lausanne). 2025 Aug 7;12:1574896. doi: 10.3389/fmed.2025.1574896 (PMC12367504; doi:10.3389/fmed.2025.1574896)
Supplement: Supplementary file 1 [file Table_1.docx]

**Supplementary Table 1** Associations between LC9 and psoriasis without arthritis based on multivariable logistic regression models.

| **LC9** | **Model 1**  **OR (95% CI)** | **Model 2**  **OR (95% CI)** | **Model 3**  **OR (95% CI)** |
| --- | --- | --- | --- |
| **Low CVH** | 1 (ref) | 1 (ref) | 1 (ref) |
| **Moderate CVH** | 1.68(0.79, 3.58) | 1.54(0.73, 3.25) | 1.45(0.71, 2.98) |
| **High CVH** | 1.63(0.73, 3.64) | 1.34(0.62, 2.88) | 1.17(0.57, 2.40) |
| **P for trend** | 0.256 | 0.594 | 0.978 |
| **Per 10-point increase** | 1.07(0.93, 1.24) | 1.03(0.89, 1.19) | 1.00(0.87, 1.14) |

Model 1: no covariates were adjusted. Model 2: sex, age, and race were adjusted. Model 3: sex, age, race, education level, PIR, marital status were adjusted. Abbreviation: LC9, Life’s Crucial 9, PIR, the ratio of income to poverty; OR, Odds Ratio, CI, Confidence Interval.
